# Web Alert: Amino acids from microbes for biotechnology: An annotated selection of World Wide Web sites relevant to the topics in *microbial biotechnology*

**DOI:** 10.1111/1751-7915.13923

**Published:** 2021-09-18

**Authors:** Lawrence P. Wackett

**Affiliations:** ^1^ Department of Biochemistry, Molecular Biology & Biophysics BioTechnology Institute University of Minnesota St. Paul MN 55108 USA

## How amino acids are made

https://www.ajinomoto.com/aboutus/amino‐acids/how‐amino‐acids‐are‐made

This website is maintained by a major company in Japan that produces amino acids via microbial fermentation processes.

## Amino acid fermentation technologies

https://www.sciencedirect.com/science/article/pii/S0734975017301052

This is a review article that focuses on the specificity of the processes used in fermentation to make amino acids via different microbial strains.

## Amino acid fermentations

https://link.springer.com/content/pdf/10.1007%2F978‐4‐431‐56520‐8.pdf

This is a book on microbial amino acid fermentations. It is comprehensive and covers the history, examples of processes, recent advances and future prospects for the field.

## Stickland fermentation

https://biocyc.org/META/NEW‐IMAGE?type=PATHWAY&object=Stickland‐Oxidative

This page of the MetaCyc database links to reactions that comprise the oxidative branch of the amino acid fermentation metabolism known as the Stickland reactions.

## Amino acid production

https://www.nature.com/articles/ja2017142.pdf?origin=ppub

This review article in the *Journal of Antibiotics* provides a very clear and concise overview of microbial amino acid production. It focuses on the microorganisms, the scale and the economic impacts of these processes.

## Production of D‐amino acids by microbes

https://www.frontiersin.org/articles/10.3389/fmicb.2018.00683/full

While most commercially relevant amino acids are in the L‐form, there is increasing evidence of the importance of D‐amino acids in the physiology of microorganisms.

## Reading amino acids in a nanopore

https://www.nature.com/articles/s41587‐019‐0345‐2

Detecting specific proteinogenic amino acids electrically is described in this report. The method can provide a new, fast way to sequence proteins.

## Amino acids from biomass

https://www.pnas.org/content/115/20/5093

Amino acids have traditionally been derived on scale via microbes that overproduce specific amino acids that they excrete into fermentation broths. This paper describes the potential to use biomass and specific catalysts to produce a number of α‐amino acids. If scalable and economically feasible, these methods could compete with the current microbial fermentation processes used in industry.

## L‐Valine production

https://www.nature.com/articles/s41598‐018‐21926‐5

This study investigated the high level production of L‐valine by *Corynebacterium glutamicum*.

## *Corynebacterium glutamicum* genomes

https://www.ncbi.nlm.nih.gov/genome/browse#!/prokaryotes/469/

*Corynebacterium glutamicum* is the single most used and useful bacterium for producing amino acids. Its genome sequence was first publicly reported in 2003, and currently, dozens of genomes of *C. glutamicum* strains have been sequenced. Links to the genomes are provided here.

## *Corynebacterium glutamicum* metabolism

https://biocyc.org/CORYNE/organism‐summary

Metabolic pathways for *Corynebacterium glutamicum* are provided here.

## Metabolic engineering of *Corynebacterium glutamicum*

https://link.springer.com/chapter/10.1007/978‐3‐030‐39267‐3_10

This review article discusses metabolic engineering of *Corynebacterium glutamicum* for enhanced amino acid production. The methods covered in this report range from classical mutagenesis, genetic engineering of single genes and systems biology approaches.

## Different microbial amino acid products

https://www.biotechnologynotes.com/amino‐acids/industrial‐production‐of‐amino‐acids‐by‐microorganism‐and‐fermentation/13820

This site provides a good basic overview of the range of amino acid products, the microorganisms used and parameters such as yield.

## Global amino acid markets

https://www.prnewswire.com/news‐releases/global‐feed‐amino‐acids‐market‐overview‐2021‐with‐profiles‐of‐56‐companies‐‐industry‐guide‐containing‐contact‐details‐for‐219‐companies‐301303039.html

This site describes the industrial demand for amino acids and the major global players in their production.

## Amino acids from *Lactococcus lactis*

https://www.sciencedirect.com/science/article/pii/S2214030120300122

This report describes a selection and bio‐sensing system to enhance the production of amino acids by *Lactococcus lactis*.

